# Revision after knee arthroplasty due to Mycoplasma hominis infection: A case report and literature review

**DOI:** 10.1097/MD.0000000000041174

**Published:** 2024-12-27

**Authors:** Kang Liu, Zhi Yang, Weipeng Xie, Sicheng Wang, Shouye Hu

**Affiliations:** a The Second Clinical Medical College, Shaanxi University of Chinese Medicine, xianyang, shaanxi, china; b Department of Joint Surgery, Hong Hui Hospital, Xi' an Jiaotong University, Xi'an, shaanxi, China; c School of Clinical Medicine, Xi'an Medical University, Xi'an, shaanxi, China.

**Keywords:** infection, Mycoplasma hominis, periprosthetic joint infection, revision, total knee arthroplasty

## Abstract

**Rationale:**

Mycoplasma hominis is an opportunistic pathogen commonly found in the human genitourinary system. However, infections caused by Mycoplasma hominis following knee arthroplasty are relatively rare.

**Patient concerns:**

A 68-year-old male patient underwent bilateral total knee arthroplasty 2 years ago due to osteoarthritis. Over the past 3 months, he developed persistent swelling and pain in both knees, along with the formation of a mass in the left knee. The patient also has a history of type 2 diabetes and hypoalbuminemia.

**Diagnoses:**

Joint fluid samples from both knees were collected for metagenomic sequencing (mNGS), which detected Mycoplasma hominis infection. Histopathological examination confirmed chronic infection.

**Interventions:**

The patient underwent 1-stage revision surgery for the left knee, followed by intravenous doxycycline (100 mg, q12h) and intra-articular injections of vancomycin (0.5 g/d) and meropenem (0.5 g/d) for 2 weeks. Afterward, the patient was switched to oral rifampin (450 mg daily) and moxifloxacin (400 mg daily) for six weeks. Following improvement in the left knee symptoms, 1-stage revision surgery was performed on the right knee. The same antibiotic regimen was used postoperatively.

**Outcomes:**

The patient experienced significant postoperative improvement, with marked pain relief and no signs of recurrent infection. The knee remained stable, and functional recovery was observed. To date, there have been no signs of infection recurrence during follow-up.

**Lessons:**

After joint arthroplasty, if a patient has persistent infection symptoms, does not respond to beta-lactam antibiotics, and has negative blood cultures, Mycoplasma infection should be considered. In this instance, the use of mNGS proved highly effective in diagnosing this atypical pathogen. The patient improved significantly after 1-stage revision surgery and targeted antibiotic therapy, though longer follow-up is needed to confirm long-term outcomes. Additionally, limited access to mNGS in some regions may delay diagnosis and treatment.

## 1. Introduction

Total knee arthroplasty (TKA) is a widely used and effective treatment for advanced osteoarthritis.^[1]^ However, periprosthetic joint infection (PJI) remains a serious complication of TKA, potentially leading to prosthetic loosening, functional impairment, and life-threatening outcomes.^[2,3]^ While PJI is most commonly caused by bacteria such as Staphylococcus aureus, rare pathogens like Mycoplasma hominis can also be involved, posing significant diagnostic challenges due to their fastidious growth characteristics.

Mycoplasma hominis, a cell wall-deficient bacterium, presents significant diagnostic challenges, as it is difficult to culture under standard conditions and often results in negative findings with conventional diagnostic methods.^[4,5]^ Metagenomic sequencing (mNGS) has emerged as a valuable tool for identifying these hard-to-culture pathogens, offering more accurate and timely diagnosis. Despite these advancements, diagnosing and treating Mycoplasma hominis-induced PJI remains difficult due to its rarity and the limited clinical experience. This report presents a case of Mycoplasma hominis-induced PJI in both knees, highlighting the challenges in diagnosis and management, and demonstrating the successful outcome following 1-stage revision surgery and targeted antibiotic therapy.

## 2. Case presentation

A 68-year-old male patient had previously undergone total knee arthroplasties on both the left and right knees at an outside hospital 2 years ago due to bilateral knee osteoarthritis, with satisfactory postoperative recovery. The patient had a history of type 2 diabetes, which was managed with long-term metformin, resulting in stable blood glucose levels. Additionally, he had hypoalbuminemia. Three months prior, the patient developed bilateral knee pain and swelling without an identifiable cause, accompanied by a noticeable mass around the left knee. He received anti-inflammatory and analgesic treatment, including cefotaxime, at a local hospital; however, the symptoms did not improve. As a result, he was referred to our hospital for further evaluation and management on April 15, 2024.

**Figure 1.**
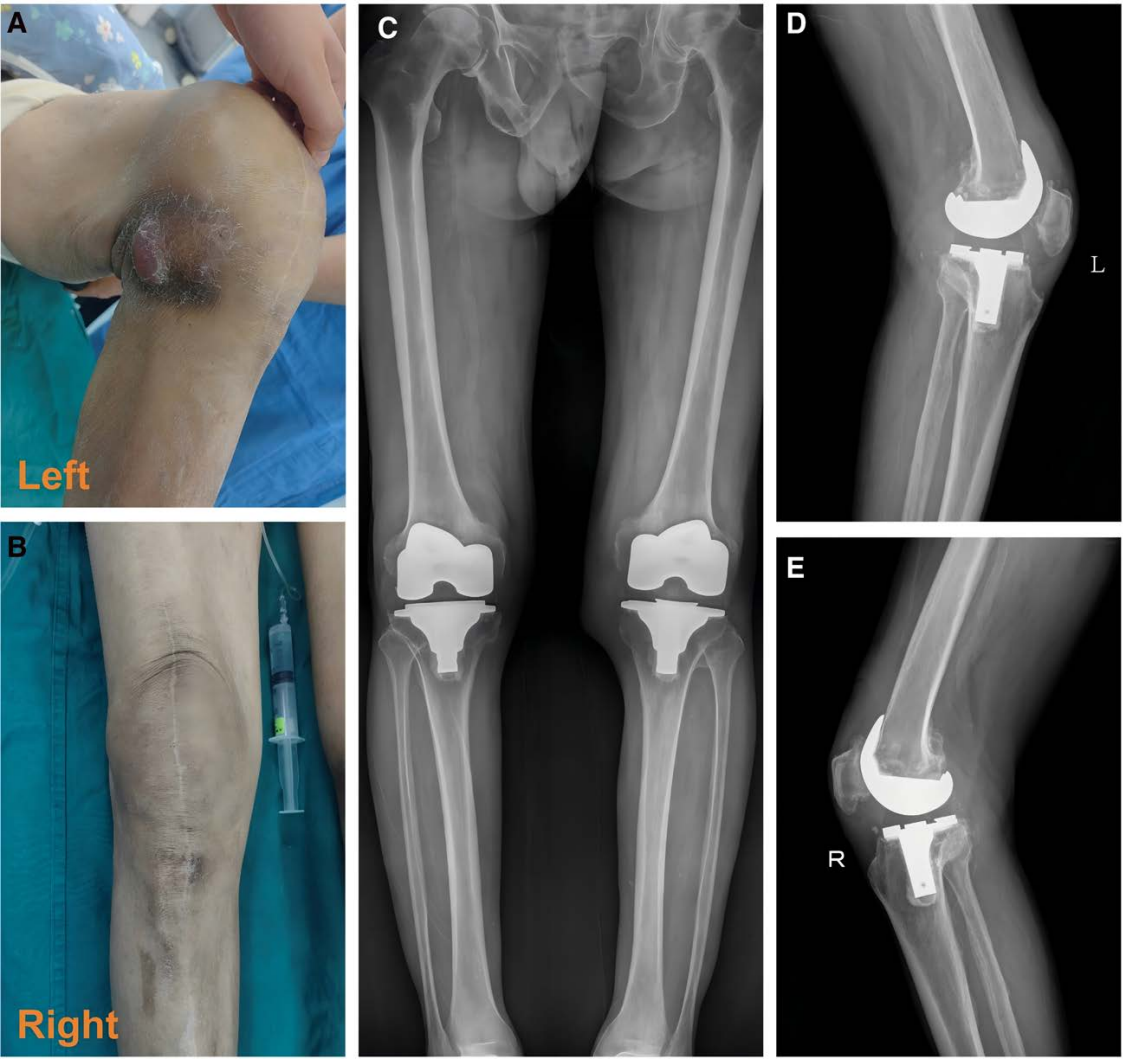
(A, B) Postoperative scars are visible on both knees, with a subcutaneous mass observed on the medial side of the left knee near the patella. (C–E) The prostheses in both knees were correctly positioned at the time of admission, with no visible radiolucent lines.

Upon admission, the patient complained of persistent knee pain, particularly in the left knee, which made it difficult for him to walk. He had no fever or chills. Physical examination revealed an 18 cm surgical scar on both knees, with a palpable subcutaneous mass on the medial side of the left knee, increased local skin temperature, and a fluctuating sensation upon palpation (Fig. 1A, B). X-rays showed proper positioning of both knee prostheses without visible radiolucency (Fig. 1C–E). Laboratory tests revealed an erythrocyte sedimentation rate of 59 mm/h (normal range: 0–22 mm/h), C-reactive protein of 30.33 mg/L (normal range: <8.0 mg/L), and albumin of 38.1 g/L (normal range: 40–55 g/L). On April 16, joint aspiration of the left knee yielded red, turbid, jelly-like synovial fluid, which was sent for testing. All cultures were negative, and empirical treatment with intravenous vancomycin (0.5g qd) was started. However, the condition did not improve, and after thorough consultation with the patient and his family, it was decided to perform revision surgery on the left knee, which was more severely affected.

On April 23, 2024, the patient underwent a left knee 1-stage revision surgery under general anesthesia with nerve block. During the procedure, the original midline incision was extended, and the sinus tract, subcutaneous mass, inflammatory granulation tissue, and purulent discharge were completely removed. Samples were sent for bacterial culture and mNGS testing. After removing the tibial liner and the femoral and tibial components, bone defects and surrounding inflammatory tissue were debrided, and the surgical area was repeatedly irrigated. After changing instruments and resterilizing, an ACCK knee system (ACCK, Beijing, China) was implanted and secured with bone cement. Knee range of motion was restored to 130°, with no dislocations or other intraoperative complications. A drainage tube was placed postoperatively, and the wound was sutured in layers with a pressure dressing. Postoperative blood cultures were negative, but mNGS, performed using the BioelectronSeq 4000 platform (GensKey, Tianjin, China), identified Mycoplasma hominis with 2173 reads. Based on this result, the patient was treated with intravenous doxycycline (100 mg, q12h) and intra-articular vancomycin (0.5 g/d) and meropenem (0.5 g/d) for 2 weeks. This was followed by oral rifampin (450 mg daily) and moxifloxacin (400 mg daily) for 6 weeks, with gradual improvement in the left knee symptoms.

**Figure 2.**
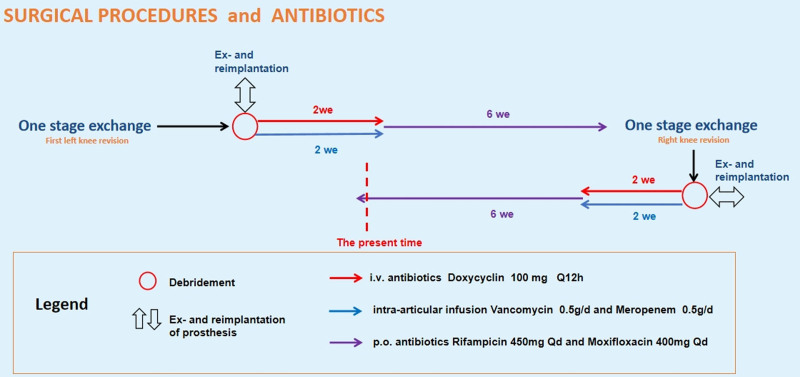
Patient diagnosis and treatment flowchart.

**Figure 3.**
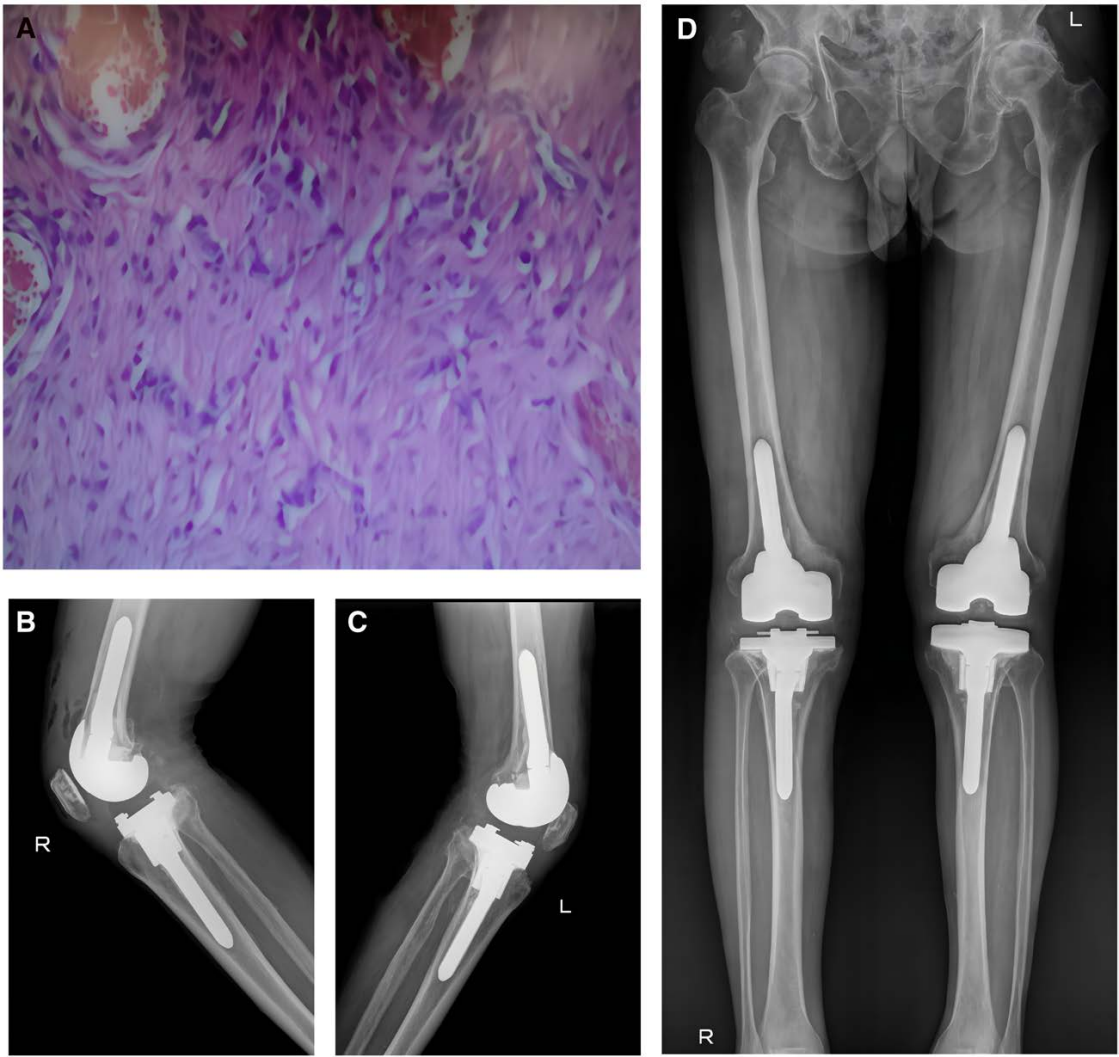
(A) Histopathological examination revealed fibrous and osseous tissue hyperplasia with chronic inflammation, hemorrhagic necrosis, sequestrum, hemosiderin deposition, and focal multinucleated giant cell reaction under light microscopy. (B) Lateral view of the right knee, (C) lateral view of the left knee, and (D) frontal view of both lower limbs showed proper alignment and positioning of the prostheses.

On May 20, joint fluid from the right knee was also sent for mNGS testing, and surprisingly, Mycoplasma hominis was detected with 1766 reads. On June 18, the patient underwent a 1-stage revision surgery on the right knee at our hospital, during which inflammatory tissue was collected for pathological examination and pathogen culture. The postoperative treatment regimen followed the same protocol as for the left knee. The treatment process is summarized in Figure 2, and the outcome was satisfactory. Postoperative cultures showed no bacterial growth. Pathological examination (Fig. 3A) of the right knee revealed fibrous and osseous tissue hyperplasia, chronic inflammation, hemorrhagic necrosis, sequestrum formation, hemosiderin deposition, and focal multinucleated giant cell reaction.Postoperative X-rays confirmed satisfactory positioning of both knee prostheses and well-corrected lower limb alignment (Fig. 3B–D). The patient recovered well and was discharged with no complications. At the latest follow-up, the patient reported good wound healing, normal lower limb function, no pain, and no signs of infection recurrence.

## 3. Discussion

Mycoplasma hominis is a common commensal organism in the genitourinary tract, capable of causing infections like urethritis, cervicitis, and pelvic inflammatory disease under certain conditions.^[5]^ However, cases of Mycoplasma hominis in PJI are extremely rare. This pathogen is primarily transmitted through sexual contact but can also spread via mother-to-child or contact transmission. In healthy individuals, Mycoplasma hominis is usually harmless as a commensal organism. However, it can cause disease in immunosuppressed individuals or those with genitourinary flora imbalance. Previous reports indicate that Mycoplasma hominis infections are more prevalent in immunocompromised individuals.^[6–9]^ This patient had type 2 diabetes and hypoalbuminemia, which likely weakened the immune system and delayed postoperative wound healing, creating favorable conditions for Mycoplasma hominis colonization and infection. Additionally, during prior surgeries, the patient may have been exposed to Mycoplasma hominis colonizing the genitourinary tract, with the use of a urinary catheter potentially serving as a transmission route. However, determining the precise source of the infection remains highly challenging.

Traditional culture methods are often ineffective in detecting Mycoplasma hominis due to its ability to form biofilms, slow growth, and lack of a cell wall, which makes it difficult to identify using Gram staining. In this case, mNGS was a critical tool for achieving a definitive diagnosis. Histopathological examination of the right knee further confirmed the chronic nature of the infection. The use of mNGS not only reduced the time required for pathogen identification but also guided the selection of appropriate antibiotics. Evidence in the literature suggests that mNGS offers significant advantages over traditional culture methods in detecting hard-to-culture pathogens, particularly those with slow growth or biofilm-forming capabilities, by allowing for earlier pathogen detection and enabling clinicians to initiate targeted antibiotic therapy sooner, which can improve patient outcomes.^[10]^ However, mNGS has limitations, including high costs and limited availability. Therefore, its use should be guided by the specific clinical context and the resources available.

Due to the absence of a cell wall in Mycoplasma hominis, conventional antibiotics such as vancomycin and β-lactams are ineffective.^[11,12]^ Consequently, tetracyclines (doxycycline) and fluoroquinolones (moxifloxacin) were selected as the primary treatment options. Previous studies have demonstrated the efficacy of doxycycline and moxifloxacin against Mycoplasma hominis infections.^[6–9,11,12]^ In this case, a combination of intravenous doxycycline and oral moxifloxacin effectively controlled the infection following surgery, with inflammatory markers significantly decreasing and no signs of recurrent infection observed during follow-up. This successful treatment highlights the importance of appropriate antibiotic selection in effectively managing Mycoplasma hominis infections. In addition, the patient underwent 1-stage revision surgery for both knees, achieving favorable clinical outcomes. However, it is important to emphasize that the choice of surgical approach should be tailored to the patient’s specific condition, taking into account factors such as the severity of the infection, the type of pathogen, the patient’s immune status, and the extent of bone and soft tissue damage.^[13–15]^ Therefore, clinicians should adopt an individualized approach to surgical planning to ensure optimal treatment outcomes.

In summary, this case highlights the need to consider Mycoplasma hominis as a potential pathogen in patients with a history of joint replacement and infections of uncertain origin, particularly those with compromised immune systems. The successful use of mNGS in this case underscores its value in diagnosing rare pathogens when conventional methods fail. Combining targeted antibiotic therapy with 1-stage revision surgery yielded favorable clinical outcomes. However, the study has limitations, including being based on a single case, the high cost and limited accessibility of mNGS, and the lack of long-term follow-up to assess recurrence risks. Despite these constraints, this case provides valuable insights for managing complex infections caused by rare pathogens, emphasizing the importance of flexible, individualized diagnostic and treatment strategies to optimize outcomes.

## Author contributions

**Conceptualization:** Shouye Hu.

**Data curation:** Kang Liu.

**Formal analysis:** Kang Liu, Shouye Hu.

**Investigation:** Kang Liu, Weipeng Xie, Sicheng Wang.

**Methodology:** Kang Liu, Shouye Hu.

**Resources:** Zhi Yang.

**Visualization:** Shouye Hu.

**Writing – original draft:** Kang Liu, Zhi Yang.

**Writing – review & editing:** Kang Liu, Shouye Hu.
